# Femoral Nerve Palsy Secondary to Iliopsoas Spontaneous Haematoma in a Patient Under Warfarin Treatment

**DOI:** 10.7759/cureus.7610

**Published:** 2020-04-09

**Authors:** Alexandros Apostolopoulos, Lefteris Kosmas, Stavros Angelis, Theodore Balfousias, Dimitrios Filippou

**Affiliations:** 1 Orthopaedics, East Surrey Hospital/Surrey and Sussex Healthcare National Health Service Trust, Redhill, GBR; 2 Orthopaedics, General Hospital Hellenic Red Cross Korgialenio-Benakio, Athens, GRC; 3 Surgical Anatomy, National and Kapodistrian University of Athens Medical School, Athens, GRC; 4 Orthopaedics, Panagiotis & Aglaia Kyriakou Children's Hospital, Athens, GRC; 5 Orthopaedics, Korgialenio-Benakio Hellenic Red Cross Hospital, Athens, GRC; 6 Surgery, National and Kapodistrian University of Athens Medical School, Athens, GRC

**Keywords:** femoral nerve, palsy, haematoma, retroperitoneal, iliopsoas muscle, warfarin, anticoagulants, vitamin k

## Abstract

Femoral nerve palsy secondary to iliopsoas non-traumatic haematoma is a scarce complication with a treatment approach that remains controversial between conservative and surgical intervention. We present a case of a 64-year-old male patient under warfarin medication, who developed severe left hip and anterior thigh pain and femoral nerve palsy with no history of trauma. Laboratory studies revealed a prolonged international normalized ratio level of 4.5, and imaging studies revealed a large haematoma surrounding the left iliopsoas muscle (35 cm x 9 cm x 6 cm). The patient was treated conservatively with discontinuation of his anticoagulation remedy and vitamin K administration and recovered almost fully after eight months, following a rehabilitation programme. Patients who are on anticoagulants should raise a high index of suspicion. Conservative management can provide a good outcome; it requires, however, a long period of rehabilitation.

## Introduction

Femoral nerve palsy due to retroperitoneal haemorrhage is an unusual complication [1-4]. In turn, retroperitoneal bleeding is most commonly the result of pelvic or lower extremity acute trauma but has also been described to develop as a less frequent complication in patients under anticoagulant therapy and bleeding disorders [1,5]. However, the incidence of femoral neuropathy secondary to spontaneous retroperitoneal haemorrhage around the iliopsoas muscle is unknown, but on the other hand, is a well-described complication of anticoagulant remedy [2,4].

There are several anticoagulant agents used in daily practice. These include warfarin, dabigatran, rivaroxaban, apixaban, acenocoumarol, and edoxaban, which are utilized in the treatment of atrial fibrillation, deep venous thrombosis, and pulmonary embolism ischaemic heart disease and for the prevention of thromboembolic events [1,2]. The use, however, of anticoagulant therapy includes high risk of haemorrhage, which is a very common complication, regularly observed in soft tissues, joints, and solid abdominal organs, in the oropharynx, or in the gastrointestinal and the genitourinary tract [2,5].

Treatment approach to these patients remains controversial [2-5]. We present a case of a male patient who was under warfarin for atrial fibrillation and developed left femoral nerve palsy, caused by a spontaneous non-traumatic left iliopsoas haematoma. The patient was treated conservatively demonstrating almost full recovery.

## Case presentation

A 64-year-old male patient was referred to the emergency department of our hospital, with a four-day history of spontaneous abdominal pain radiating on the posterior aspect of the left thigh. He reported no trauma history, but during the past few hours, pain had severely deteriorated. In regards to his past medical history, the patient was on treatment for hypertension and atrial fibrillation. In particular, he was on an angiotensin II receptor blocker and on warfarin as an oral anticoagulant. 

The patient was afebrile, and his vital signs were within normal values (blood pressure 115/78 mmHg, heart rate 82 beats/min, SpO2 99%). Physical examination revealed diffuse abdominal pain, radiating on the posterior aspect of the left thigh. Peripheral vascular examination was within normal values on both lower limbs, with palpable pulses and capillary refill time of less than two seconds. Neurological condition, on the other hand, was impaired on the left lower limb, and objective deficiencies were obvious. Examination revealed reduced strength of the left lower limb. The patient was unable to straight leg raise, to flex the hip or the knee, and had developed hypoesthesia on the anterior aspect of the thigh. The patella reflex was absent on the affected side.

**Figure 1:**
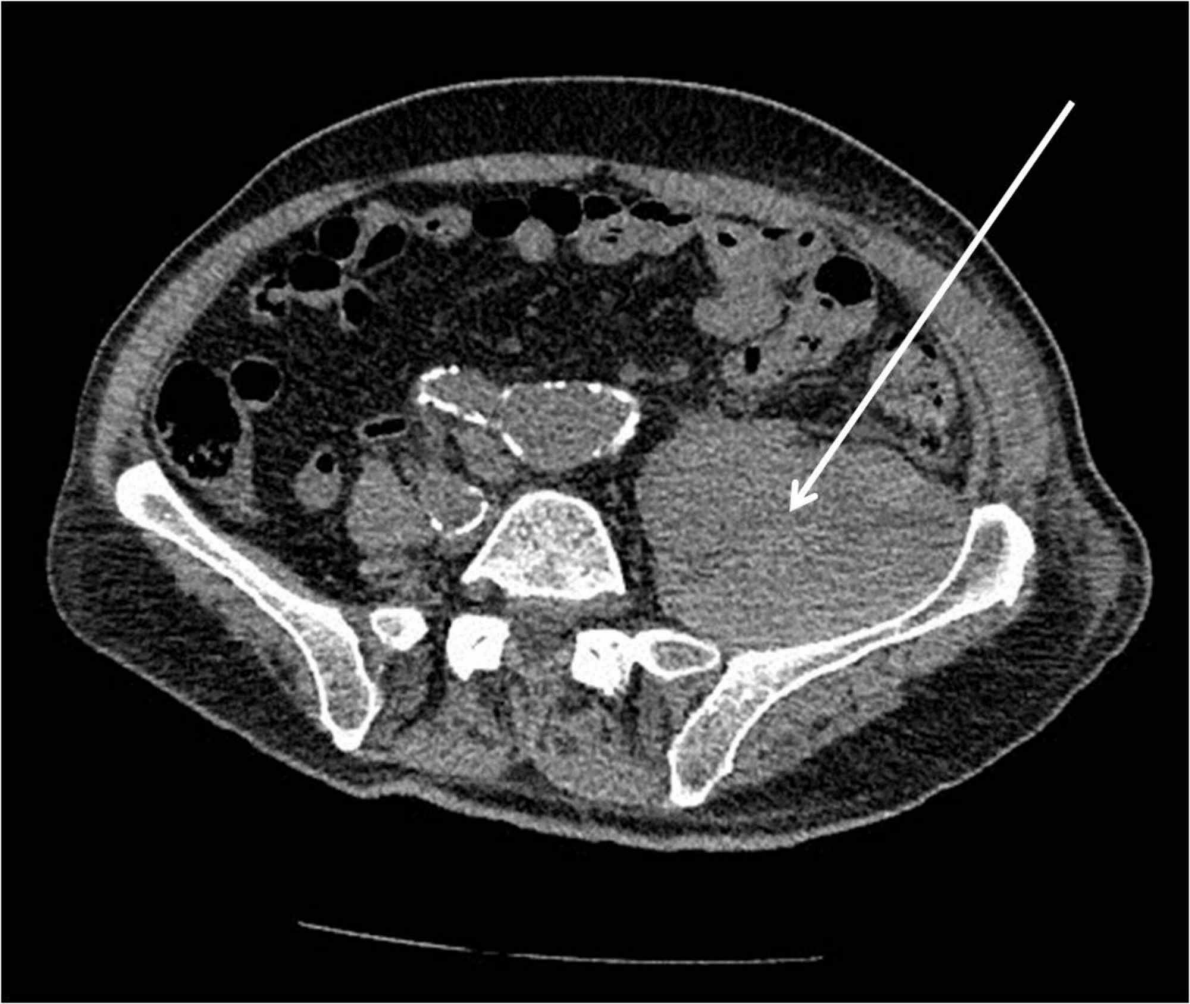
CT scan revealing a left iliopsoas haematoma (arrow) on day of admission.

Laboratory data revealed prolonged prothrombin time with an international normalized ratio (INR) level of 4.5, and a haematocrit and haemoglobin decrease of 33.1% and 10.7 g/l, respectively. The rest of the full laboratory analysis was within normal values. Imaging studies included abdominal ultrasound scan (US), lumbar spine, and pelvis CT scans. Lumbar spine pathology was excluded, and a large retroperitoneal haematoma was unveiled within and around the left iliopsoas muscle (35 cm x 9 cm x 6 cm) (Figure 1).

**Figure 2:**
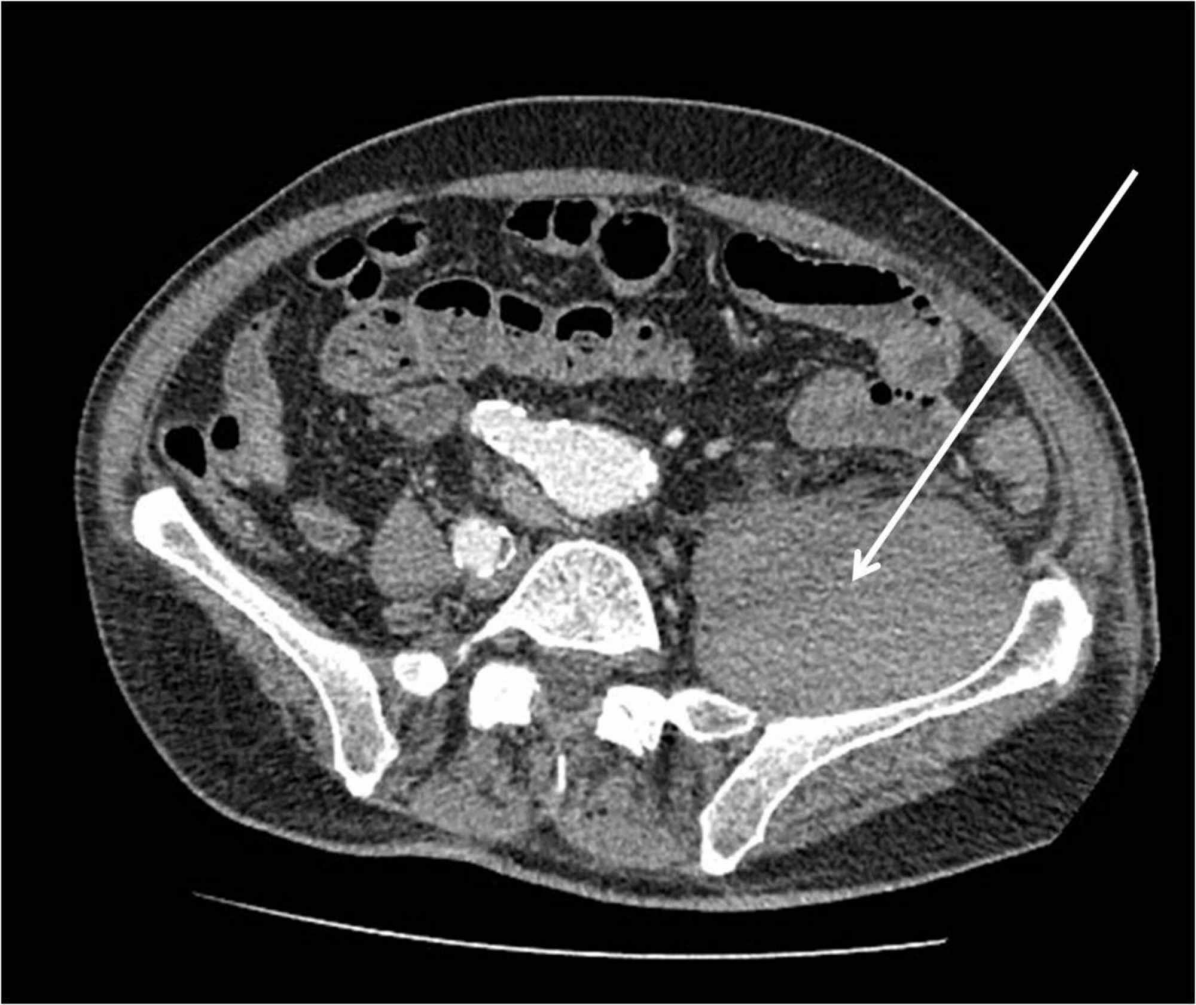
CT scan performed on day 3 post-admission did not reveal any significant change in regards to the size of the haematoma (arrow).

The patient was admitted to the hospital, and warfarin anticoagulation regimen was intermitted. Prolonged INR was reversed within 48 hours with the aid of vitamin K administration. A new CT scan was performed on day 3 post-admission. No change in the size of the haematoma was revealed (Figure 2). At that point, there was a consultation with the radiology department whether the haematoma could be percutaneously drained; however, the CT scan revealed a clotted formation that would not allow for successful drainage.

**Figure 3:**
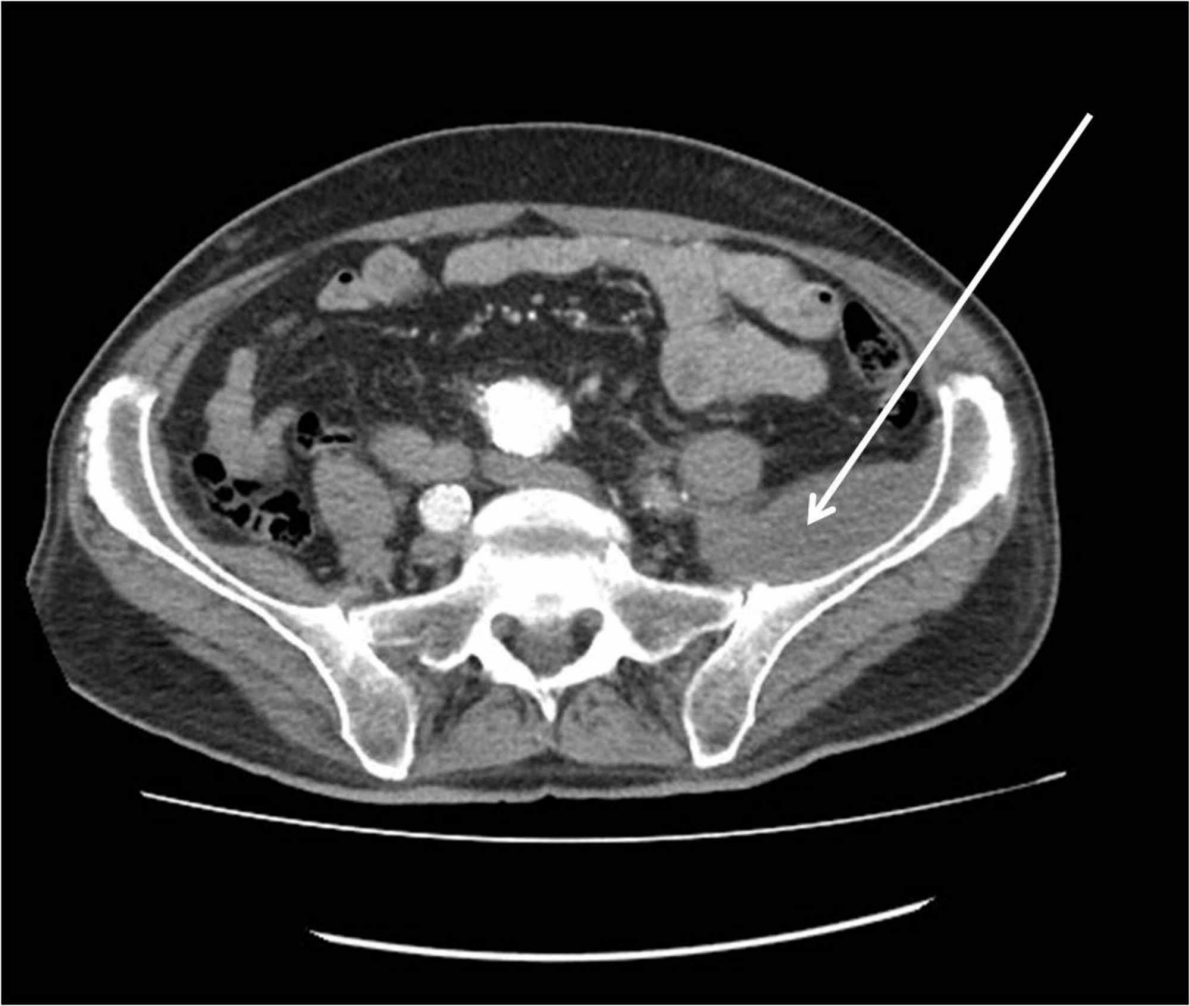
CT scan performed two months post-discharge revealing absorption and a significantly reduced in size haematoma (arrow).

The patient was managed conservatively. Physical therapy had been initiated by day 1 post-admission. The patient was discharged after six days of hospitalization and after pain management had been established. Nerve conduction studies were performed three weeks later and revealed left femoral nerve palsy. A new CT scan was performed two months post-discharge and unveiled absorption and a significantly reduced size of the haematoma (Figure 3). The patient demonstrated gradual improvement and recovered after eight months of physiotherapy and rehabilitation. This included almost complete motor and complete sensory function restoration of the left femoral nerve.

## Discussion

Retroperitoneal haemorrhage is most commonly encountered as a result of pelvic acute trauma and can be a life-threatening situation [1,5]. Spontaneous haemorrhage of the iliopsoas muscle followed by femoral nerve neuropathy is a rare complication frequently seen in patients who are on anticoagulants [1,2,5]. The neuropathy is a result of the decreased blood flow to the surrounding epineurium or it can befall secondary to the pressure-related ischaemia [1,2]. The diagnosis is suspected by the patient’s past medical history and by a thorough clinical and neurological examination and should be confirmed by CT scan [5].

Conservative management of this includes discontinuation of the anticoagulation therapy and reversal of any coagulopathy [3]. In our case, the patient was on warfarin treatment. The most common complication of warfarin is bleeding, and INR is recommended that should be monitored regularly [2,6]. The INR of our patient was prolonged at time of admission (INR 4.5). Several medications and various dietary products can affect INR levels [6]. The effective half-life of warfarin is between 20 and 60 hours (mean 40 hours) [6]. Medication adverse effects may be reversed with phytomenadione (vitamin K1), fresh-frozen plasma, and prothrombin complex concentrate [6]. Approximately 5 mg of vitamin K were administered orally on day 1 and 2.5 mg on day 2 post-admission. INR levels decreased to 2.7 and dropped to 1.6 on day 3 post-admission.

The best treatment approach remains a matter of controversy. Conservative management is discouraged by some researchers in severe cases [1,2-5]. Some authors suggest that the haematoma should be percutaneously drained under CT or US guidance in order to reverse the pressure-induced nerve ischaemia [2-5]. This possibility was addressed to our radiology department but was soon rejected, as it was suggested that the haematoma was already organized and a CT aspiration would, therefore, be ineffective. Open surgical debridement has also been described as a managing option depending on the severity of the femoral neuropathy and the size of the haematoma [1,4,5]. In cases of haemodynamic instability, blood transfusion and crystalloid administration may be required [7].

Prognosis is usually good, and most patients, treated operatively or not, recover and return to their previous functionality [5]. Our patient recovered eight months post-discharge, regaining almost 100% of his muscle strength and complete sensory function by avoiding surgical intervention, even though CT depiction and neurological deficiency implied severe femoral nerve pressure, at time of admission.

## Conclusions

Femoral nerve palsy secondary to iliopsoas non-traumatic haematoma is a scarce complication. History and full neurological examination are imperative to suspect the diagnosis. Patients who are on anticoagulants should raise a high index of suspicion, and a CT scan should be performed. Conservative management can provide a good outcome; it requires, however, a long period of rehabilitation.
